# What are the long-term patient-reported and clinical outcomes after lateral clavicle fractures? A cross-sectional study of 619 patients

**DOI:** 10.1007/s00068-022-02062-2

**Published:** 2022-08-04

**Authors:** Rens A. van der Linde, Svenhjalmar van Helden, Sarah Woltz, Mostafa El Moumni, Frank F. A. IJpma

**Affiliations:** 1 Department of Surgery, University Medical Center Groningen, University of Groningen, Hanzeplein 1, 9713GZ Groningen, The Netherlands; 2 Department of Surgery, Isala Hospital Zwolle, Zwolle, The Netherlands; 3 Department of Surgery, Spaarne Gasthuis, Haarlem, The Netherlands

**Keywords:** Clavicle fracture, Operative, Conservative, Quality of life

## Abstract

**Background:**

Lateral clavicle fractures account for 17% of all clavicle fractures and large studies comparing nonoperative and operative treatment are lacking. Therefore, patients cannot be properly informed about different treatment options and prognosis. We assessed long-term patient-reported and clinical outcomes in patients with lateral clavicle fractures.

**Methods:**

A multicenter cross-sectional study was performed in patients treated for lateral clavicle fractures between 2007 and 2016. Primary outcome included patient-reported outcome measures (PROMs) (DASH, EQ-5D, return to work, sports, cosmetics and satisfaction). Questionnaires were sent to 619 eligible patients, of which 353 (57%) responded after a mean follow-up of 7.4 ± 2.8 years. Secondary outcome included adverse events and secondary interventions. Outcomes after nonoperative vs. operative treatment (stratified by nondisplaced vs. displaced fractures) were compared using Student* t* tests and linear regression analysis.

**Results:**

Nondisplaced lateral clavicle fractures were treated nonoperatively and resulted in excellent PROMs. Six patients (3%) developed a nonunion. For displaced lateral clavicle fractures, no differences were found between nonoperative and operative treatment with regard to DASH score (7.8 ± 12.5 vs 5.4 ± 8.6), EQ-5D (0.91 ± 0.13 vs 0.91 ± 0.09), pain (0.9 ± 1.7 vs. 0.8 ± 1.6), patient satisfaction (90.1 ± 25.5 vs. 86.3 ± 20.4), return to work (96.4% vs. 100%) and sports (61.4% vs. 62.3%). The absolute risk of nonunion in patients with a displaced fracture was higher after nonoperative than operative treatment (20.2% vs. 2.9%; *p* = 0.002), with six patients needing treatment to avoid one nonunion.

**Conclusions:**

Nondisplaced lateral clavicle fractures should be treated nonoperatively and result in good functional outcomes and high union rates. For displaced fractures, neither nonoperative nor operative treatment seems superior. Patients opting for nonoperative treatment should be informed that nonunion occurs in 20% of patients, but only half of these need additional operative treatment. Patients who opt for surgery should be told that nonunion occurs in only 3%; however, most patients (56%) will require secondary intervention for elective implant removal. Regardless of the type of treatment, no differences in functional outcome and PROMs should be expected at long-term follow-up.

**Supplementary Information:**

The online version contains supplementary material available at 10.1007/s00068-022-02062-2.

## Introduction

### Background

Occurring predominantly in both young male and elderly patients, the annual incidence of clavicle fractures is estimated between 29 and 64 per 100.000 [1–3]. The majority of fractures is situated in the middle third (81%), followed by the lateral (17%) and medial third (2%) of the clavicle [2, 4]. Lateral clavicle fractures can be treated either operatively or nonoperatively. The choice of treatment depends on multiple factors including fracture displacement and involvement of the coracoclavicular ligaments [5]. Most non- or minimally displaced fractures can safely be treated with appropriate pain management, a sling and physical therapy [6, 7]. In contrast, displaced fractures can be unstable and may benefit from operative treatment. A variety of surgical techniques have been proposed each with its pros and cons, including multiple fracture fixation devices (i.e., hook plate, lateral clavicular plate, tension band wiring and transacromial pinning with Kirschner wires) and coracoclavicular fixation techniques (i.e., tight rope or endobutton and screw) [8].

### Rationale

There is no consensus about the optimal treatment strategy of lateral-third clavicle fractures. Decisions to treat lateral clavicle fractures operatively or nonoperatively and which operative technique to use are still unclear. Most literature on clavicle fractures is focused on midshaft fractures. A recent systematic review reported on 22 RCTs of midshaft clavicle fractures and found 88% union rate after nonoperative vs. 97% after operative treatment with no substantial difference in functional outcome at 1-year follow-up [9]. However, results of midshaft fractures are not automatically generalizable to lateral-third fractures, because involvement of the coracoclavicular ligaments and the close relationship with the AC-joint. No large studies have been performed to compare nonoperative, operative and specific surgical treatments for lateral clavicle fractures. High-quality studies with long-term follow-up are lacking, rendering it difficult to guide patients in making an informed treatment decision [10, 11].

### Research questions

We therefore asked, in patients with nondisplaced vs. displaced lateral clavicle fractures (1) what are the long-term patient-reported outcomes in terms of physical functioning, health-related quality of life, pain, return to work, sports, cosmetics and overall satisfaction? (2) What is the clinical outcome in terms of adverse events and secondary interventions?

## Patients/Methods

### Study design

After obtaining a waiver from the local Medical Ethical Review Boards (METc 2018.00809 and 2018.180809) a cross-sectional study was performed. All consecutive patients who received either nonoperative or operative treatment for a lateral clavicle fracture at two level one trauma centers between 2007 and 2016 were eligible for inclusion.

### Study population

**Fig. 1:**
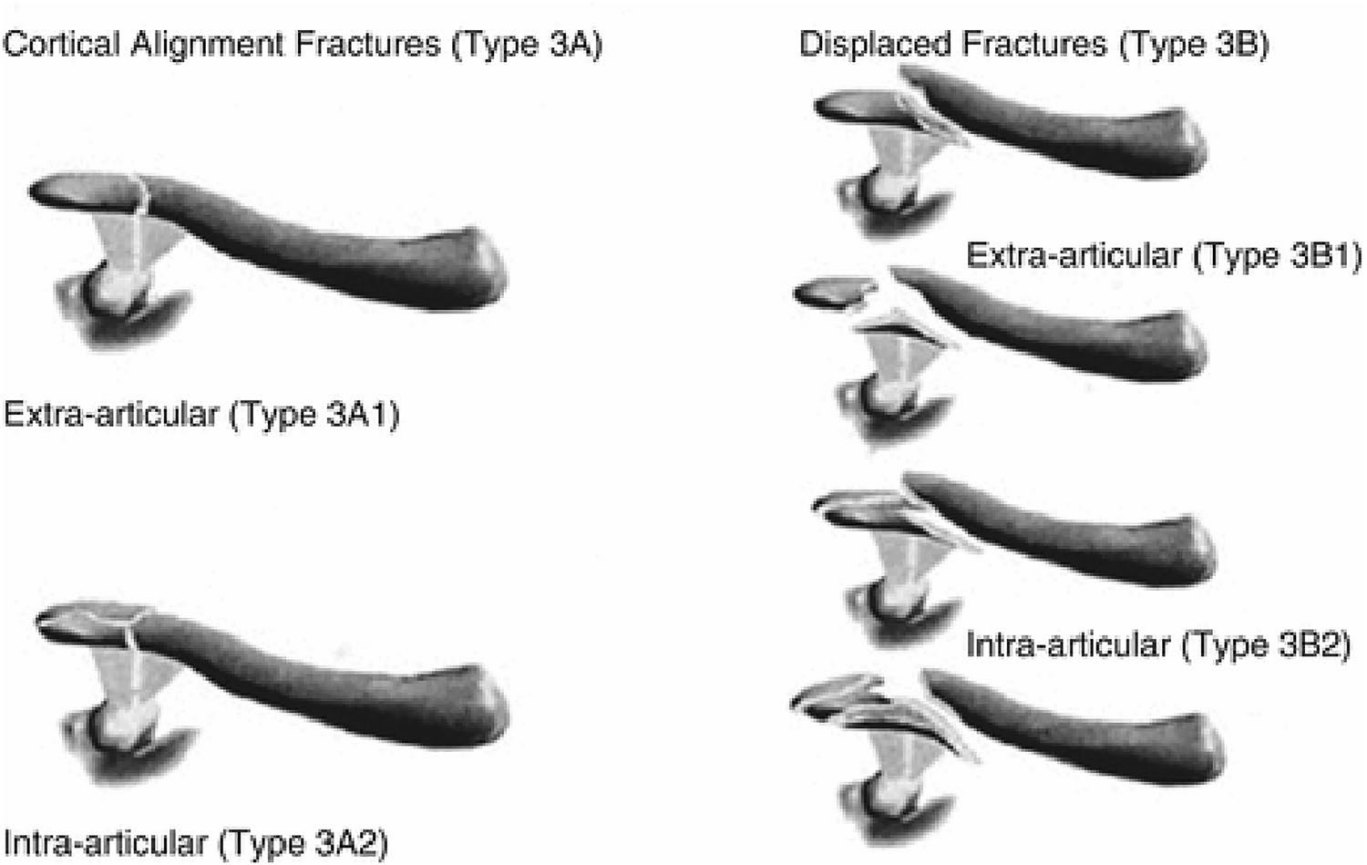
Robinson classification

Lateral clavicle fractures were defined as fractures of the lateral third of the clavicle (type 3A and 3B according to the Robinson classification; Fig. 1) [3]**.** A displaced fracture was defined as a fracture with more than one shaft width displacement. Nondisplaced or minimally displaced fractures were treated nonoperatively. Fracture displacement (e.g., defined as a fracture with more than one shaft width displacement) was an indication for recommending operative treatment. Some patients with a displaced fracture might not have had surgery due to shared decision-making. In case of operative treatment, the choice for the type of operative technique was according to the preference and expertise of the treating surgeon. Included were all patients aged > 15 years who sustained a lateral clavicle fracture.

### Treatment and outcome

Hospital records were reviewed to acquire patient characteristics and information concerning the course of treatment and complications. All radiographs were reassessed to determine fracture classification, displacement and union rate. Nonoperative treatment consisted of immobilization, using a sling, followed by mobilization exercises combined with appropriate analgesic management. Surgical reports were reviewed to determine the type of implant used for fixation. For all patients, the national population registry was contacted to verify their current address and to be sure they were not deceased. Patients were approached by posted mail and asked to complete and return a set of validated patient-reported outcome measures.

The primary outcome measure for this study was patient-reported outcome in terms of (1) physical functioning, (2) health-related quality of life (QoL), (3) pain, return to (4) work, (5) sports, (6) cosmetic and (7) overall satisfaction. The ‘Disability of the Arm, Shoulder and Hand’ (DASH) questionnaire was used to measure physical functioning, with scores ranging from 0 (no disability) to 100 (most severe disability) [12–15]. QoL was assessed using the ‘EQ-5D-5L’ (EuroQol Group, Rotterdam, The Netherlands) questionnaire, which consists of five questions, and a visual analogue scale (VAS) assessing the patient’s self-reported health [16]. Patients were asked to rate their cosmetic and overall satisfaction with the treatment using a visual analogue scale (VAS) from 0 (not satisfied at all) to 100 (completely satisfied). Pain was assessed using a numeric rating scale (NRS) ranging from 0 (no pain) to 10 (worst pain imaginable) and two additional questions concerning the use of pain medication. Return to work and sport were assessed by two additional questions. Secondary outcomes in this study were clinical outcomes in terms of (8) adverse events (i.e., complications) and (9) secondary operations. Information concerning secondary outcomes was acquired from patient files and two additional questions in the follow-up questionnaires.

### Statistical methods

Descriptive statistics were used to analyze patient characteristics. Results were presented using mean and standard deviation (SD). For the primary and secondary outcome, a Student *t* test was used to compare the means of parametric independent variables. Linear regression was used to analyze the relationship between the (operative vs. nonoperative) treatment of displaced lateral clavicle fractures and the DASH score. First, a crude model was computed. A second model was constructed to adjust for potential confounders, including gender, age at the time of injury, trauma mechanism, dominant arm injured, complications, work and sport. Patients with missing data were excluded from further analysis. Outcomes are presented as mean, followed by the SD and 95% confidence interval (CI) in parentheses. All executed tests were two-sided, results with *P* < 0.05 were considered statistically significant.

All eligible patients with lateral clavicle fractures between 2007 and 2017 were eligible for inclusion in order to acquire a true representation of the general patient population and a sufficient follow-up time of at least 3 years. Post hoc power analysis revealed that with *α* = 0.05%, a sample size of 136 patients, 68 in each group, was required to provide 90% power to detect a difference of 10 points for DASH score, with a standard deviation of 18. A difference of 10 points in DASH is considered the minimal clinically important difference (MCID) [17, 18]. Statistical analyses were performed using SPSS software, version 23 (IBM Corp **©**, Armonk, New York, USA).

## Results

### Participants

**Fig. 2:**
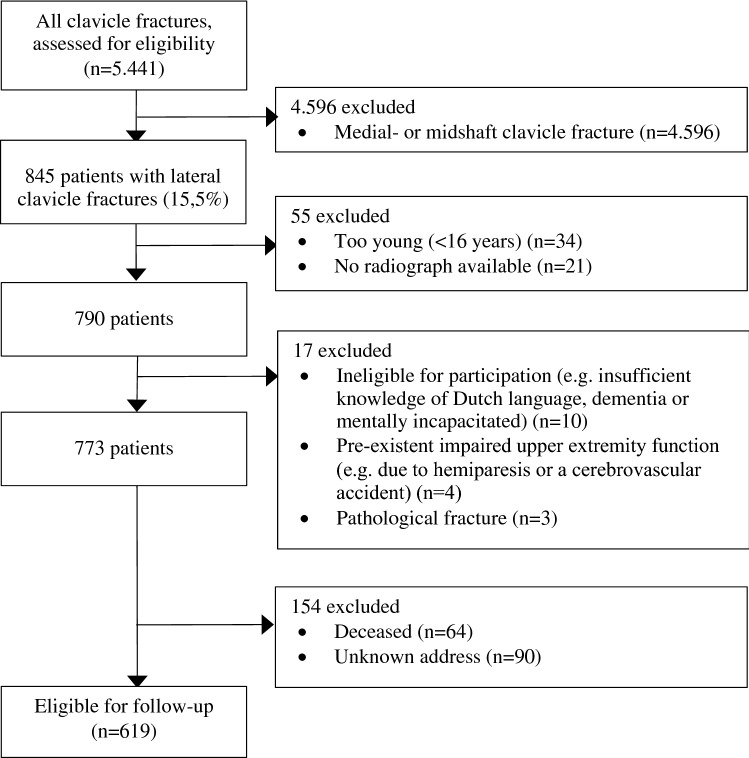
Flowchart of the included patients

Between 2007 and 2016, a total of 845 patients were treated for lateral clavicle fracture, of which 619 were eligible for follow-up analyses with patient-reported outcome measures (also see Fig. 2). Questionnaires concerning clinical outcomes were sent by posted mail to 619 patients, of whom 353 (response rate 57%) responded with a mean follow-up of 7.4 (SD 2.9) years after the initial injury. Nineteen patients refused participation, three patients returned incomplete questionnaires and were therefore excluded from further analyses. Baseline characteristics of the included patients are shown in Table 1. A nonresponse analysis was performed which showed a difference at the time of injury in mean age between responders (45.9 ± 15.7 years) and nonresponders (38.1 ± 15.5; *P* < 0.001) and sex (responders 66.9% male vs. nonresponders 76.3% male; *p* = 0.013) (Table 4, supplementary file). There were no differences in fracture type, kind of treatment and duration of follow-up.

**Table 1:** Baseline characteristics of included patients with a lateral clavicle fracture (*N* = 331)

	Nondisplaced (*N* = 179)	Displaced (*N* = 152)	All patients (*N* = 331)	*p* value
Sex				
Male	111 (62.0)	114 (75.0)	225 (68.0)	0.016*
Female	68 (38.0)	38 (25.0)	106 (32.0)	0.016*
Age at time of injury mean (SD)	46.0 (16.7)	45.7 (14.3)	45.9 (15.6)	0.836
Trauma mechanism^†^				
Traffic	104 (58.4)	101(66.9)	205 (62.3)	0.143
Sports	25 (14.0)	25 (16.6)	50 (15.2)	0.633
Fall	38(21.3)	18 (11.9)	56 (17.0)	0.034*
Fall from height	4 (2.2)	4 (2.6)	8 (2.4)	1.000
Other	7 (3.9)	3 (2.0)	10 (3.0)	0.483
Dominant arm	75 (41.9)	69 (45.4)	144 (43.5)	0.598
Robinson classification				
3A1	153 (85.5)	0 (0)	153 (46.2)	< 0.001*
3A2	26 (14.5)	0 (0)	26 (7.9)	< 0.001*
3B1	0 (0)	134 (88.2)	134 (40.5)	< 0.001*
3B2	0 (0)	18 (11.8)	18 (5.4)	< 0.001*
Initial treatment				
Nonoperative	179 (100)	84 (55.3)	263 (79.5)	< 0.001*
Operative	0 (0)	68 (44.7)	68 (20.5)	< 0.001*
Hook plate	0 (0)	20 (29.4)	20 (6.0)	NA
Superior plate	0 (0)	36 (52.9)	36 (10.9)	NA
Tension band wiring	0 (0)	9 (13.2)	9 (2.7)	NA
CC augmentation + superior plate	0 (0)	3 (4.4)	3 (0.9)	NA
Hospital admission in days mean (SD)	0.4 (1.7)	1.2 (2.3)	0.8 (2.1)	< 0.001*
Follow-up in years mean (SD)	7.5 (2.9)	7.2 (3.0)	7.3 (3.0)	0.323

Data are expressed in *N* with percentages in parentheses unless otherwise specified

*p < 0.05 was considered to be significant

^†^Data not available for 2 patients (one in each group)

### Initial treatment

All nondisplaced or minimally displaced fractures were treated nonoperatively. Regarding displaced fractures, 68 out of the 152 (44.7%) fractures were treated operatively (Table 1). Surgery was performed after a median of 9 days (interquartile range 6–13 days) after injury. The most frequently used surgical technique was plate osteosynthesis using either a superior plate (52.9%) or hook plate (29.4%). Other techniques included tension band wiring (11.8%) and a superior plate with coracoclavicular (CC) augmentation using a tight rope (4.4%).

### Patient-Reported outcomes

There was no difference in functional outcome between nonoperative and operative treatment for lateral clavicle fractures after a mean follow-up of 7.3 ± 3.0 years (DASH 7.6 ± 13.9 vs. 5.4 ± 8.6; *p* = 0.115). Subgroup analysis of displaced fractures revealed no difference in functional outcome between nonoperatively treated and operatively treated fractures (DASH 7.8 ± 12.5 vs. 5.4 ± 8.6, respectively; *p* = 0.175). When the analysis was adjusted for potential confounding factors, a mean difference of 4.0 (95% CI 0.5–7.4; *p* = 0.025) in DASH score was found in favor of operatively treated group.

All patients reported a good Quality of life (QoL), represented by high scores on the EQ-5D-5L questionnaire regardless of treatment type (Table 2). There was no difference in QoL between nonoperative and operative treatment (0.92 ± 0.14 vs. 0.92 ± 0.09; *p* = 0.774). Subgroup analysis of patients with a displaced fracture, revealed no difference in QoL between nonoperative and operative treatment (0.91 ± 0.13 vs. 0.92 ± 0.09, respectively; *p* = 0.505).

**Table 2:** Patient-reported outcomes after treatment for lateral clavicle fractures (*N* = 331)

	Nonoperative nondisplaced (*N* = 179)	Nonoperative displaced (*N* = 84)	Operative displaced (*N* = 68)	Total population (*N* = 331)	Nonoperative vs. operative (*p* value)	Nonoperative displaced vs. operative displaced (*p* value)
DASH	7.5 (14.6)	7.8 (12.5)	5.4 (8.6)	7.2 (13.0)	0.115	0.175
EQ-5D-5L	0.92 (0.14)	0.91 (0.13)	0.92 (0.09)	0.92 (0.13)	0.774	0.505
Pain						
In rest	0.6 (1.6)	0.9 (1.7)	0.8 (1.6)	0.7 (0.1)	0.796	0.771
With movement	0.9 (2.0)	1.1 (2.3)	0.9 (1.7)	1.0 (2.0)	0.817	0.551
Use of painkillers (%)	8 (4.5)	3 (3.6)	1 (1.5)	12 (3.6)	0.482	0.768
Overall satisfaction	87.5 (19.1)	80.1 (25.5)	86.3 (20.4)	85.4 (21.3)	0.687	0.098
Cosmetic satisfaction	88.7 (21.7)	75.4 (27.3)	79.6 (21.8)	83.5 (23.9)	0.136	0.304
Work (%)						
Unemployed/retired pre-injury	66 (36.9)	29 (34.5)	11 (16.2)	106 (32.0)	0.002*	0.018*
Employed pre-injury	113 (63.1)	55 (65.5)	57 (83.8)	225 (68.0)	0.002*	0.018*
Return to work	110 (97.3)	53 (96.4)	57 (100)	220 (97.8)	0.425	0.460
Sport (%)						
No sport pre-injury	33 (18.4)	16 (19.0)	8 (11.8)	57 (17.2)	0.247	0.317
Sporting actively pre-injury	146 (81.6)	68 (81.0)	60 (88.2)	274 (82.8)	0.247	0.317
Partial return to sport	26 (17.8)	19 (27.9)	10 (16.7)	55 (20.1)	0.573	0.191
Full return to sport	106 (72.6)	43 (63.2)	38 (63.3)	187 (68.2)	0.442	1.000

Data are expressed in mean with SD in parentheses unless otherwise specified

*P < 0.05 was considered to be significant

Low levels of pain both at rest (0.7 ± 1.6 vs. 0.8 ± 1.6; *p* = 0.796) and with movement (1.0 ± 2.1 vs. 0.9 ± 1.7; *p* = 0.817) were found in the nonoperative as well as in the operative group. Hence, very few patients reported use of painkillers on a daily basis (4.2% vs. 1.5%; *p* = 0.482). Subgroup analyses of displaced fractures showed no differences in either pain at rest (0.9 ± 1.7 vs. 0.8 ± 1.6; *p* = 0.771) or with movement (1.1 ± 2.3 vs. 0.9 ± 1.7; *p* = 0.551) and use of painkillers (3.6% vs. 1.5%; *p* = 0.768) between nonoperative and operative treatment.

There were no differences in both overall satisfaction with treatment (85.1 ± 21.6 vs. 86.3 ± 20.4; *p* = 0.687) and satisfaction with the cosmetic result (84.5 ± 24.4 vs. 79.6 ± 21.8; *p* = 0.136) between nonoperative treatment and operative treatment (Table 2). Furthermore, subgroup analyses revealed no differences in overall (80.1 ± 25.5 vs. 86.3 ± 20.4; *p* = 0.098) and cosmetic satisfaction (75.4 ± 27.3 vs. 79.6 ± 21.8; *p* = 0.304) in patients who received nonoperative or operative treatment for a displaced lateral clavicle fracture.

Patients who received operative treatment were pre-injury more commonly employed compared to nonoperatively treated patients (84% vs. 64%; *p* = 0.002). After the injury and subsequent treatment, both the nonoperative and operative group reported excellent return to work rates (97% vs. 100%; *p* = 0.425). Subgroup analysis of patients with a displaced fracture revealed no differences in return to work between nonoperatively and operatively treated patients (96% vs.100%; *p* = 0.460). There were no substantial differences in engagement in sport pre-injury between patients who received nonoperative or operative treatment (90% vs. 81%; *p* = 0.129). Post-injury, both the nonoperative and operatively treated patients reported comparable return to sport rates (partial return to sport 21% vs. 16.7%; *p* = 0.573 and full return to sport 69.6% vs. 63.3%; *p* = 0.442, respectively). Subgroup analysis of the nonoperatively displaced group compared with the operatively displaced group showed no differences in return to sport (partial return to sport 27.9% vs 16.7; *p* = 0.191 and full return to sport 63.2% vs. 63.3%; *p* = 1.000, respectively).

### Clinical outcome

Perioperative complications included one (1.5%) fracture-related infection [19] and two (2.9%) implant failures, for which early implant removal was performed (Table 3). One patient who developed a fracture-related infection was treated with surgical debridement, implant revision and subsequent antibiotics. In general, union was achieved in 97.1% of the operatively treated patients and 91.3% of the nonoperatively treated patients (*p* = 0.039). Subgroup analyses of displaced fractures showed a higher nonunion rate after nonoperative treatment than after operative treatment (20.2% vs. 2.9%; *p* = 0.002), the number needed to treat to avoid one nonunion was 5.84. Almost half (47.1%) of the initially nonoperatively treated patients who developed a nonunion underwent a secondary operation for nonunion treatment with plate fixation. On the other hand, more than half of patients (54.4%) after primary operative treatment underwent a secondary operation for elective implant removal (16 out of 20 (80%) of hook plates, 13 out of 36 (33%) of superior plates and 8 out of 8 (100%) of tension band wiring).

**Table 3:** Clinical outcomes in patients treated for a lateral clavicle fracture (*N* = 331)

	Nonoperative nondisplaced (*N* = 179)	Nonoperative displaced (*N* = 84)	Operative displaced (*N* = 68)	Total population (*N* = 331)	Nonoperative vs. operative	Nonoperative displaced vs. operative displaced
Adverse events (%)						
Fracture-related infection	0 (0)	0 (0)	1 (1.5)	1 (0.3)	NA	NA
Implant failure	0 (0)	0 (0)	2 (2.9)	2 (0.6)	NA	NA
Nonunion	6 (3.4)	17 (20.2)	2 (2.9)	25 (7.6)	0.039*	< 0.001*
Secondary operations (%)						
Infection treatment	0 (0)	0 (0)	1 (1.5)	1 (0.3)	NA	NA
Nonunion treatment	6 (3.4)	17 (20.2)	2 (2.9)	25 (7.6)	0.039*	< 0.001*
Conservative	1 (16.7)	9 (52.9))	2 (100)	10 (3.0)	NA	NA
Resection	2 (33.3)	0 (0)	0 (0)	2 (0.6)	NA	NA
Plate fixation	3 (50)	8 (47.1)	0 (0)	11 (3.3)	NA	NA
Elective implant removal	0 (0)	0 (0)	37 (54.4)	35 (10.6)	NA	NA

Data are expressed in *N* with percentages in parentheses unless otherwise specified

*p < 0.05 was considered to be significant

## Discussion

### Background and rationale

Current literature mainly focuses on clavicle shaft fractures, and large series concerning patient-reported and clinical outcomes for patients with lateral clavicle fractures are lacking [9]. We performed a cross-sectional study representing the largest cohort of patients with lateral clavicle fractures to date. This study demonstrates that nondisplaced lateral clavicle fractures can with confidence be treated nonoperatively resulting in excellent patient outcomes with high union rates (97%) at long-term follow-up. Displaced lateral clavicle fractures can either be treated nonoperatively or operatively. Operative treatment yields higher union rates compared to nonoperative treatment (97% vs. 80%), with approximately 6 patients who need to be operated to avoid one nonunion. However, half of patients with a nonunion was asymptomatic and did not require additional surgical treatment. On the other hand, approximately 50% of patients after primary operative treatment required a second operation for elective implant removal. There were no differences in functional outcome, quality of life, patient satisfaction, return to work and sport between nonoperative and operative treatment of displaced lateral clavicle fractures at long-term follow-up.

### Patient-reported outcomes

When comparing nonoperative and operative treatment for lateral clavicle fractures, no differences in patient-reported functional outcome and quality of life were found at a mean follow-up of 7.3 ± 2.9 years. Adjusting for potential confounding factors using linear regression, we found a significant difference in DASH scores of 4.0 (95% CI 0.5–7.4; *p* = 0.025) between nonoperatively and operatively treated patients with displaced factures. This finding did not meet the level of a minimal clinically important difference and was therefore deemed clinically irrelevant [17, 18, 20]. Furthermore, there were no differences in pain, return to work, sport and patient satisfaction. Hall et al. performed the only available randomized controlled trial comparing nonoperative and operative treatment in 57 patients with acute displaced lateral clavicle fracture [21]. They found no differences in functional outcome at any point in time up to one year after the initial treatment. In addition, they found no differences between the two groups regarding return to work or other activities. However, the sample size of this study was limited and the a priori calculated simple size was not reached. This makes it difficult to draw definitive conclusions, and the authors advocate further research in order to determine which treatment provides the most desirable results. A recent retrospective study by Kihlström et al. reporting on 122 patients with lateral clavicle fractures found no differences in patient-reported outcome between operatively and nonoperatively treated patients after a median follow-up of 3.1 (IQR 2.3–4.2) years, which seems in line with our results. However, our study adds to these findings, because in their study only 30 out of 122 (25%) patients were available for follow-up analysis compared to 353 out of 619 (response rate 57%) patients with mean follow-up of 7.4 (SD 2.9) years in our study [22].

Different operative techniques have been described for the stabilization of displaced lateral clavicle fractures, each with their pros and cons [23–29]. A recent systematic review, comparing different operative treatment strategies in patients with displaced lateral clavicle fractures, found that plate fixation was associated with the highest postoperative function scores and lowest risk of complications, followed by coracoclavicular fixation [8]. They suggested that plate fixation should be the first choice, in terms of safety and efficacy, for treatment of displaced lateral clavicle fractures. Another retrospective study by Hickland et al. reported on different operative treatment strategies in 44 patients with displaced clavicle fractures [26]. Patients were treated by either hook plate fixation, locking plate fixation, locking plate fixation and coracoclavicular ligament reconstruction (CCLR) or CCLR alone. They found that, besides high rates of implant removal after hook plate fixation, none of the treatment modalities was superior. However, comparison with a control group of nonoperatively treated patients was lacking in most case series or small cohorts that report on surgical techniques [24–29]. Robinson et al. reported that nonoperative management in 101 patients with a displaced lateral clavicle fracture resulted in good functional outcome and quality of life after a mean follow-up of 6.2 years [6]. The results of these studies endorse our findings that, at the long-term, both nonoperative and operative treatment may result in a good functional outcome and comparable results in terms of pain, return to work, sport activities and patient satisfaction.

### Clinical outcomes

It has been reported that nonoperative treatment of displaced lateral clavicle fractures is associated with substantial nonunion rates [21, 30, 31]. In 2004, Robinson et al. reported a 21% nonunion rate after nonoperative treatment, although only 14% eventually opted for a delayed surgical intervention [6]. This nonunion rate is equivalent to our results (20.2%). The rate of secondary interventions for nonunions is comparable between their and our study (14% vs. 9.5% of the whole cohort). Operative treatment for lateral clavicle fractures enables early mobilization, reduced pain and earlier return of function, but also carries the risk of surgery-related complications [23, 32–34]. Adverse events are uncommon and may vary between the different treatment options [35]. Complications associated with operative treatment may include fracture-related infection, implant failure, impingement and degenerative acromioclavicular alterations [8, 36, 37].

With only one fracture-related infection (1.5%) and two implant failures (2.9%) in this study, operative treatment could be considered a safe treatment option for patients with displaced lateral clavicle fractures. Furthermore, operative treatment for displaced clavicle fractures yielded a high union rate of 97% in the present study. This is in line with union rates reported in the literature, ranging from 93 to 100% [35]. Even though operative treatment considerably reduces the rate of nonunions (3%), it was also associated with a high risk (54.4%) of a secondary intervention for elective implant removal. This is consistent with reintervention rates in recent literature [21, 22].

### Limitations

We acknowledge that selection bias is inherent to cross-sectional cohort studies, caused by either referral or selective loss to follow-up and nonresponse [38, 39]. We tried to reduce the risk of selection bias by approaching all eligible patients. Moreover, the response rate in our study was high (57%) and nonresponse analysis only revealed a small difference in age (45.9 ± 15.7 vs 38.1 ± 15.5 years; *p* < 0.001) between the responders and nonresponders. Another limitation is that this study does not provide longitudinal data about short-term patient outcomes. This information could be an important consideration for active, high-demanding patients. Moreover, due to the limited number of patients in the different treatment groups comparison between the different operative treatment strategies was not feasible. Last, we acknowledge that missing data about adverse events or complications might occur in cross-sectional studies. However, the risk of missing data concerning adverse events and complications was considered low, because it was acquired from patient files as well as from additional questions in the follow-up questionnaires.

## Conclusion

This present study shows that nondisplaced lateral clavicle fractures can safely be treated nonoperatively with excellent functional outcome, quality of life and high union rates (97%) at long-term follow-up. For displaced lateral clavicle fractures, neither nonoperative nor operative treatment seems superior. There were no differences in functional outcome, quality of life, pain, return to work, sport and patient satisfaction between nonoperative and operative treatment at long-term follow-up. The definitive choice of treatment should be weighted between an increased absolute risk of a nonunion (20% vs. 3%) after nonoperative treatment against the high chances of undergoing a second procedure (54%) for elective hardware removal after primarily operative treatment. Half of the patients with a nonunion did not opt for additional surgical treatment. These findings from our multicenter cross-sectional study can be used as a guideline toward personalized treatment and shared decision-making in the management of displaced lateral clavicle fractures. Further studies, either large randomized controlled trials or prospective observational studies where natural variation in treatment allocation is utilized, are needed to assess clinical outcome after nonoperative vs. operative treatment (including comparison of different surgical techniques) of displaced lateral clavicle fractures.

## Supplementary Information

Below is the link to the electronic supplementary material.

Supplementary file1 (DOCX 15 kb)
