# Supplementary material for: What are the long-term patient-reported and clinical outcomes after lateral clavicle fractures? A cross-sectional study of 619 patients
Source: Eur J Trauma Emerg Surg. 2022 Aug 4;49(1):289–98. doi: 10.1007/s00068-022-02062-2 (PMC9925498; doi:10.1007/s00068-022-02062-2)
Supplement: Supplementary file 1 — Supplementary file1 (DOCX 15 kb) [file 68_2022_2062_MOESM1_ESM.docx]

| **TABLE IV. Baseline characteristics nonresponse analysis (N=619)** | | | | |
| --- | --- | --- | --- | --- |
|  | **Responders (N=353)** | **Nonresponders (N=266)** | **All patients (N=619)** | **P value** |
| **Sex (%)** |  |  |  |  |
| Male | 236 (66.9) | 203 (76.3) | 439 (70.9) | 0.013* |
| Female | 117 (33.1) | 63 (23.7) | 180 (29.1) | 0.013* |
| **Age at time of injury** mean **(SD)** | 46.8 (15.8) | 38.1 (15.4) | 43.2 (16.3) | <0.001* |
| **Fracture classification (Robinson; %)** |  |  |  |  |
| 3A1 | 168 (47.6) | 139 (52.3) | 307 (49.6) | 0.286 |
| 3A2 | 27 (7.6) | 19 (7.1) | 46 (7.4) | 0.934 |
| 3B1 | 140 (39.7) | 90 (33.8) | 230 (37.2) | 0.161 |
| 3B2 | 18 (5.1) | 18 (6.8) | 36 (5.8) | 0.481 |
| **Initial treatment (%)** |  |  |  |  |
| Non-operative | 283 (80.2) | 219 (82.3) | 502 (81.1) | 0.497 |
| Operative | 70 (19.0) | 47 (17.7) | 117 (18.9) | 0.497 |
| Hookplate | 20 (24.7) | 6 (11.5) | 26 (19.5) |  |
| Superior plate | 50 (61.7) | 34 (65.4) | 84 (63.2) |  |
| Tension band wiring | 8 (9.9) | 10 (19.2) | 18 (13.5) |  |
| Resection | 2 (2.5) | 2 (3.8) | 4 (3.0) |  |
| Other | 1 (1.2) | 0 (0) | 1 (0.8) |  |
| **Delayed union (%)** | 9 (2.5) | 2 (0.8) | 11 (1.8) | 0.171 |
| **Nonunion (%)** | 25 (7.1) | 14 (5.3) | 39 (6.3) | 0.450 |
| **Follow-up in years** mean (SD) | 7.4 (2.9) | 7.4 (2.7) | 7.4 (2.8) | 0.877 |
| Data is expressed in N with percentages in parentheses unless otherwise specified. * P<0,05 was considered to be significant | | | | |
